# Hyper-IgE syndrome: a case report

**DOI:** 10.1097/MS9.0000000000001670

**Published:** 2024-01-03

**Authors:** Tirth Dave, Fatema Ali Asgar Tashrifwala, Umme Salma Rangwala, Rizwanullah Hameed

**Affiliations:** a Bukovinian State Medical University, Chernivtsi, Ukraine; b Department of Research and Discovery Stamford Health, Stamford, CT; c Mahatma Gandhi Memorial Medical College, Indore, India; d Interfaith Medical Centre, New York, NY

**Keywords:** elevated IgE levels, hyper-IgE syndrome, job syndrome, primary immunodeficiency

## Abstract

**Introduction and importance:**

Hyper-IgE syndrome (HIES), also known as Job syndrome, is a rare primary immunodeficiency disorder characterized by elevated serum IgE levels, recurrent infections, and various clinical features. Early diagnosis, prompt management of infections, and supportive care are essential in improving outcomes for individuals with HIES. Genetic testing, including STAT3 gene sequencing, plays a crucial role in confirming the diagnosis. Further research is needed to enhance our understanding of HIES and develop targeted therapies to improve the quality of life for affected individuals.

**Case presentation:**

This case report presents the clinical features and management of a 37-year-old male with HIES, diagnosed at the age of 2 due to recurrent cold abscesses caused by Staphylococcal infections.

**Clinical discussion:**

The patient exhibited typical symptoms of HIES, including recurrent eczema, frequent bacterial infections, mucocutaneous candidiasis, and various physical abnormalities. Diagnostic markers such as elevated IgE levels and eosinophilia supported the HIES diagnosis, which was further confirmed by the identification of a STAT3 gene mutation. Treatment primarily involved supportive measures and antibiotics for infections. The patient’s blood test results and imaging findings revealed abnormalities such as low red blood cell count, elevated erythrocyte sedimentation rate, and pulmonary nodules.

**Conclusion:**

This case report highlights the importance of early diagnosis, prompt management of infections, and the need for ongoing research to improve our understanding and treatment of HIES.

## Introduction

Highlights

- Hyper-IgE syndrome has only 250 documented cases worldwide.
- Genetic testing, including STAT3 gene sequencing, plays a crucial role in confirming the diagnosis.
- Our patient is a 37-year-old male with a history of hyper-IgE syndrome, diagnosed at 2 years of life due to recurrent episodes of cold abscesses on his back and groyne caused by a Staphylococcal infection. IgE levels were 10 000 IU/ml.

Previously known as Job’s syndrome, hyper-IgE syndromes (HIES) are a heterogeneous group of inborn errors of immunity. The underlying cause of HIES is dominant-negative (DN) mutations in various signal transduction and activators of transcription genes. The most well-studied form, referred to as STAT3-HIES (OMIM #102582), is associated with mutations in the STAT3 gene located on chromosome 17^1^. Additionally, there have been reports of other forms of HIES resulting from mutations in different genes. These include HIES2 (OMIM #243700), which arises from mutations in the DOCK8 gene, HIES3 (OMIM #618282), associated with mutations in the ZNF341 gene, HIES4A (OMIM #619752) and HIES4B (OMIM #618523), both stemming from mutations in the IL6ST gene (600694), and HIES5 (OMIM #618944), caused by mutations in the IL6R gene^1^. The incidence of this disease is less than 1 per million population, and until now only 250 cases have been documented worldwide. The first case of elevated IgE level was recognized as a cardinal feature of the syndrome in 1972, and the name HIES was subsequently proposed. Also, in 2007, autosomal dominant mutations in signal transducer and activator of transcription-3 (STAT3) gene were identified as the molecular cause of this disease.

Clinical features include recurrent eczema, frequent bacterial infections, and mucocutaneous candidiasis. Skin rash usually appears in the first week of life, recurrent pneumonia and cold abscess with staphylococcus aureus are common. The patient can present with non-healing skin eruptions. Characteristic facial features that typically appear in early adolescence or late childhood include asymmetry, a prominent forehead, deep-set eyes, a broad nasal bridge, a wide fleshy nasal tip, rough facial skin, an increased inter-alar distance, prognathism, and a high-arched palate. Birth defects observed in individuals with HIES include craniosynostosis, a condition where the sutures of the skull fuse prematurely, and Arnold Chiari type 1 malformation, which involves the displacement of the cerebellar tonsils into the spinal canal. Musculoskeletal abnormalities observed encompass joint hyperextensibility, scoliosis, and osteopenia. Decreased bone density contributes to the occurrence of numerous pathological fractures, primarily affecting long bones and ribs, reported in ~50% of patients. Retention of three or more primary teeth has been documented in around 70% of individuals with Job syndrome. Vascular manifestations include tortuosity or dilation of blood vessels, as well as the presence of aneurysms in coronary, cerebral, and aortic regions, along with congenital abnormalities in the coronary arteries^2^.

Diagnosis of HIES is typically based on clinical suspicion. Key diagnostic markers include a total IgE concentration exceeding 1000 IU/ml and eosinophilia, which are observed in over 90% of patients. The gold standard for confirming a HIES diagnosis is the STAT3 Gene Sequencing test, which detects mutations in the STAT3 gene and allows for the identification of a mutant STAT3 protein. Additionally, the HIES Screen by Flow test assesses the normal phosphorylation of STAT3 in patient cells following cytokine stimulation and measures the presence of interleukin-17 (IL-17)-producing TH17 cells within the CD4+ T-cell population. To further support the diagnosis, physicians may request chest X-rays or computed tomography (CT) scans to evaluate the presence of pneumatoceles, as well as a bone density scan to assess bone health^3^. Differential diagnoses can be HIV or chronic granulomatous diseases. However, it is often associated with non-Hodgkin’s lymphoma, myocardial infarction, lacunar infarcts, hypertension, systemic lupus erythematosus, and glomerular nephritis. Treatment is symptomatic and inter-professional and aims to treat the complications. Bone marrow transplantation can be considered for treatment as well as stem cell therapy if a suitable donor is available. Research is underway on dupilumab, an antagonist of the IL-4 receptor α chain to treat this rare syndrome^4^.

## Case presentation

**Figure 1:**
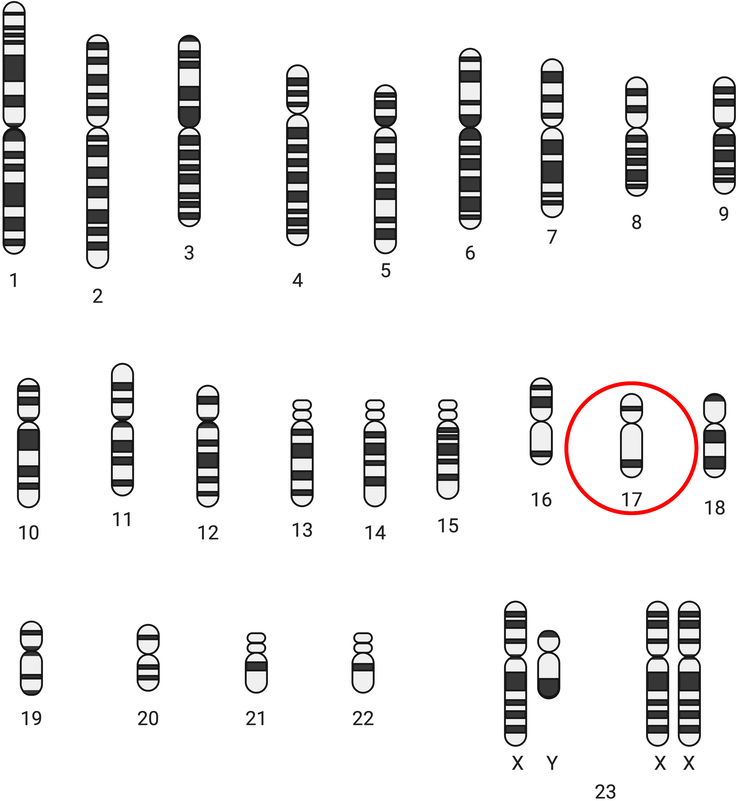
Karyotype of the patient (original figure by the authors).

**Figure 2:**
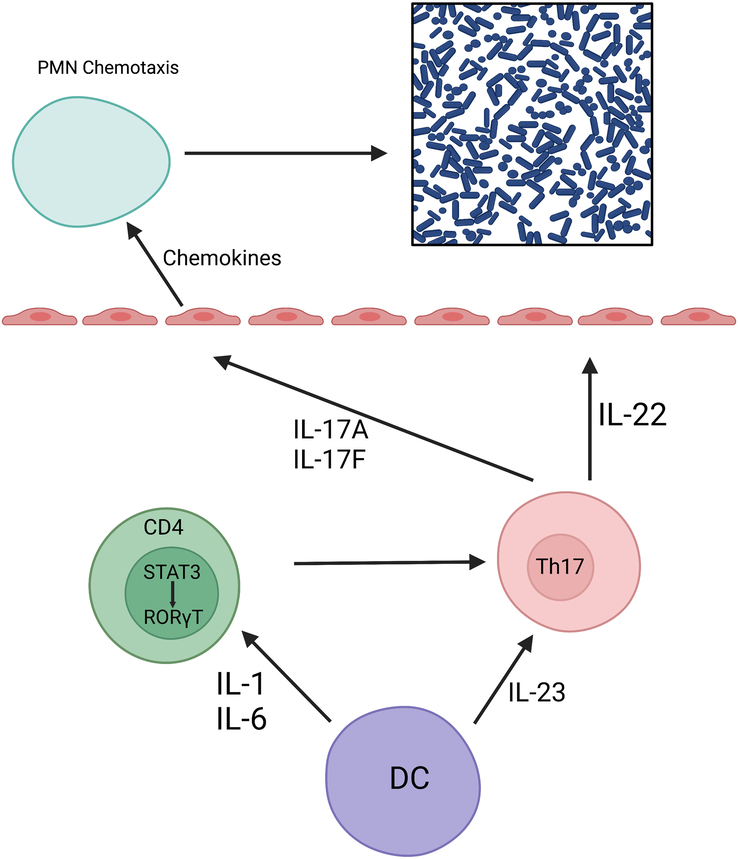
Representation of pathway of how hyper-IgE syndrome increases susceptibility to infections (original figure by the authors). IL, interleukin.

We present the case of a 37-year-old male with a history of HIES, diagnosed at 2 years of life due to recurrent episodes of cold abscess on his back and groyne caused by a Staphylococcal infection. The patient belonged to an under-resourced area and had limited access to healthcare as well a lack of awareness in parents resulted in the delay in diagnosis. Also, physicians provided symptomatic care in the earlier years of life as this diagnosis being rare was not considered. IgE levels were 10 000 IU/ml with eosinophilia. In this case, a bone marrow biopsy was not performed. The patient was not born to consanguineous parents and the mother denies any use of alcohol, drugs, or tobacco during pregnancy. The patient has had multiple cold abscesses since then, however, only three have required incision and drainage. The patient was started only on supportive treatment and antibiotics, nothing specific for HIES. The karyotype of the patient is presented in Figure 1 and the pathway through which HIES increases susceptibility to infections is presented in Figure 2.

Past medical history was significant for multiple hospitalizations, rapid weight loss, hyperpigmentation on the skin, autism and Down syndrome, dermatitis, and Globulinemia that is HIES. The patient was administered the following medications: ascorbic acid, ferrous sulfate, megestrol acetate (for weight gain), and tylenol.

The patient first presented to the infectious disease physician in 2012 because of abscesses on both of his buttocks. He was already on IV antibiotics which he accidentally removed. On physical examination, hyperpigmentation, rash on the face, groyne, and buttocks, and neurological examination were indicative of autism and Down syndrome, while the rest of the physical examination was normal. The patient was repeatedly removing the IV antibiotic line by accident.

In 2013, the patient presented with an abscess accompanied by foul-smelling oozing pus, along with fever and chills. They experienced a similar abscess presentation again in 2014 and 2016. Starting in 2017, the patient began to have a loss of bowel and bladder control, necessitating the use of adult diapers. In 2018, they presented with fever and chills attributed to multiple cysts and rhinitis.

In 2019, the patient presented with a dry cough and was prescribed new medications, including azithromycin 500 mg, promethazine 6.25 mg/5ml, augmentin 500–125 mg, and miconazole nitrate for a fungal infection. They also started taking ergocalciferol. Follow-up visits with an infectious disease physician occurred in 2020. In 2021, the patient sought a dose adjustment of Hydrocortisone tablets, leading to a gradual tapering of the 20 mg dose. They were advised to continue taking 5 mg of Hydrocortisone on a long-term basis.

In 2022, the patient presented with swelling in the right armpit, which was not accompanied by fever or pain. Upon examination, a 5×7 cm lesion was identified in the right axilla and diagnosed as an abscess-draining yellow discharge with a foul odour. Additional findings included pressure ulcers stage II over bilateral buttocks, which were also draining yellow discharge with a foul odour. The patient completed antibiotic therapy and reported poor appetite, resulting in the initiation of Megestrol to promote weight gain.

After a few months, the patient returned with a draining sinus located at the sacrum and was initiated antibiotic treatment. Subsequently, the patient has been receiving regular follow-up care through telehealth services. It is important to note that the patient belongs to an underserved population and is actively seeking clinical trials that could provide additional treatment options for IgE-related conditions. Due to limited available information, the available reports mainly consist of blood test results, chest X-ray findings, and cardiology reports. HIES was diagnosed using the Invitae Hyper IgE Syndrome Panel, which revealed a STAT3 gene mutation. The diagnosis was supported by the patient’s clinical features and serum IgE levels of 10,000 IU.

The blood test results and clinical abnormalities are identified and summarized in Table 1.

**Table 1:** Blood test results and clinical abnormalities.

Test	Result	Interpretation	Reference range	Previous results	Units
RBC count	3.72	Low	4.20–5.80	3.48	MILL/MCL
WBC count	10.2	Normal	3.80–10.40	11.4	THOUS/MCL
Neutrophils	7.7	High	1–6.20	9.1	THOUS/MCL
Lymphocytes	1.3	Normal	1– 4.30	1.2	THOUS/MCL
Monocytes	0.8	Normal	0.10–1	0.8	THOUS/MCL
Eosinophils	0.4	Normal	<0.8	0.2	THOUS/MCL
Basophils	0.1	Normal	<0.2	0.1	THOUS/MCL
MCH	21.1	Low	27–33	22.5	PG
MCV	71.4	Low	80–100	73.2	FL
ESR	134	High	<10	137	MM/HR
Haemoglobin	7.9	Low	13.20–17.10	7.8	PL
Haematocrit	26.5	Low	38–50	25.5	%
MCHC	29.6	Low	32–36	30.7	G/dl
TIBC	244.7	Low	255–450	—	mg/dl
Iron	13	Low	50–212	—	ug/dl
Iron %saturation	5.3	Low	15–50	—	%
Anisocytosis	1+	Normal	—	2+	—
Polychromasia	1+	Normal	—	—	—
Giant platelets	Few	Normal	—	Present	—

%, percentage; ug/dl, micrograms per decilitre; ESR, erythrocyte sedimentation rate; FL, femtoliter; G/dl, grams per decilitre; MCH, mean corpuscular haemoglobin; MCHC, mean corpuscular haemoglobin concentration; MCV, mean corpuscular volume; mg/dl, milligrams per decilitre; MILL/MCL, millions per microliter; MM/HR, millimetres per hour; PG, picogram; PL, platelet count; RBC count, red blood cell count; THOUS/MCL, thousands per microliter; TIBC, total iron binding capacity; WBC count, white blood cell count.

The chest X-ray revealed pulmonary peri-bronchial thickening and the presence of multiple small nodules in the right lung. No signs of focal pulmonary consolidation, pleural effusion, pneumothorax, or cardiomegaly were observed. Hyperinflation of the lung fields was noted, along with mild scoliosis. The presence of very small nodular infiltrates in the right lung confirms the diagnosis of bronchitis or pneumonitis. Cardiac echo showed a normal left ventricular ejection fraction of 55–60%, and mild thickening of the anterior and posterior mitral valve leaflets.

## Discussion

HIES, also known as Job syndrome, belongs to a group of primary immunodeficiency diseases characterized by a unique combination of elevated IgE levels, recurrent pulmonary and cutaneous infections, and eczematous rashes. While HIES is typically sporadic, there have been reports of genetic inheritance patterns including autosomal dominant, autosomal recessive, and X-linked modes^5^.

The first reported case of HIES was presented by David *et al.* ^6^. Two patients exhibited recurrent pulmonary infections, eczema, and multiple skin abscesses. These abscesses were unique as they lacked the typical signs of inflammation such as redness, pain, and warmth, and were therefore referred to as “cold” abscesses. At the time, there was no association made between these symptoms and elevated levels of IgE, as immunoglobulin had not yet been discovered^7^. Subsequent case reports by Buckley *et al.*, Hill *et al.* ^8^, and Grimbacher *et al.* ^9^. further identified additional manifestations of HIES, including dysmorphic facial features, markedly high serum IgE levels, and dental and connective tissue abnormalities. Due to its rarity, with ~250 documented cases, accurately estimating the prevalence of HIES is challenging, as it is often misdiagnosed or underdiagnosed^10^.

An aetiologically significant cause of HIES is dominant-negative (DN) mutations in the signal transducer and activator of the transcription-3 (STAT3) gene. The loss-of-function (LOF) mutations in the Dedicator of cytokinesis 8 (DOCK8) gene are responsible for the autosomal recessive form of HIES. STAT3 is a transcription factor that binds to the promoter region of various genes such as that cytokine, leukaemia inhibitory factor, corticotrophin, oncostatin, receptor tyrosine kinases, insulin-like growth factor-1, growth hormone, etc. Therefore, STAT3 plays an important role in cell survival and inflammation in tissues of the skin, lungs, thymus, mammary glands, and of cells such as macrophages, neurons, and lymphocytes. A majority of STAT3 mutations are missense mutations and occur in the DNA binding domain. These mutations result in impaired production of acute-phase proteins due to a compromised interleukin-6 (IL-6) response in the liver^7^.

Autosomal dominant HIES (AD-HIES) presents during infancy with recurrent bacterial and fungal infections caused by Staphylococcus aureus, Streptococcus pneumoniae, Hemophilus influenzae, Candida, and Aspergillus. The cutaneous infection presents as a pustular rash affecting the face and scalp, scrapings of which reveal eosinophilia. The onset of eczema is also during infancy, which remains to be the only allergy-like symptom. Recurrent pulmonary pyogenic infections are complicated by the development of pneumatoceles and bronchiectasis, which subsequently serve as a nidus for fungal and other opportunistic infections. Further dissemination of causative pathogens is a major cause of mortality. Unlike other primary immunodeficiency diseases, AD-HIES also affects organ systems other than the immune system^5,7^. Extra-immunologic manifestations include facial features such as a prominent forehead, increased intercanthal distance, primary teeth retention, osteoporosis, recurrent pathologic fractures, scoliosis, and hyperextensible joints. Arnold Chiari 1 malformation and craniosynostosis may also appear in some patients with AD-HIES. Vascular abnormalities may be seen in adults with AD-HIES such as myocardial infarction, coronary artery tortuosity, dilations, and aneurysms. Patients are also increasingly susceptible to malignancies like lymphomas^5,11^.

The autosomal recessive form of HIES (AR-HIES), is distinct from AD-HIES in that it does not present with non-immunologic features like abnormal facies, milk teeth retention, etc. In this variant, there is a higher incidence of viral infections affecting the skin, such as those caused by herpes simplex (HSV) and molluscum contagiosum. Mortality due to sepsis and central nervous system afflictions like infections and vasculitis are more common in AR-HIES than in AD-HIES^11^.

HIES is mainly diagnosed using clinical presentation and laboratory findings. Serum IgE levels are elevated in all patients with HIES and levels can typically reach as high as 100 000 IU ml. The serum levels of immunoglobulins G, A, and M remain normal. Eosinophilia of greater than or equal to 700 cells μl is seen in most cases. Based on the presence and severity of 21 clinical and laboratory findings, the NIH scoring system was developed specifically for diagnosing AD-HIES^5^.

Treatment options are few, mainly supportive treatment with antibiotics for cutaneous infections and antibiotic prophylaxis for pulmonary infections. The goal of therapy should be to prevent these infections, and if they occur, to aggressively treat them to prevent the development of complications that may lead to mortality.

## Conclusions

In conclusion, HIES is a rare primary immunodeficiency disorder characterized by recurrent infections, elevated IgE levels, and a range of clinical features. The disease stems from genetic mutations that disrupt the immune system’s ability to combat infections and regulate inflammation. Patients with HIES may experience various symptoms, including skin infections, pneumonia, respiratory infections, bone and joint abnormalities, and dental issues.

Despite its rarity, understanding and accurately diagnosing HIES are crucial for effective management and treatment. Treatment approaches typically involve a combination of preventive measures such as prophylactic antibiotics, antifungal drugs, and immunoglobulin replacement therapy. Early detection and prompt treatment of infections are vital to effectively manage the disease. Immunology research has contributed to advancing our comprehension of HIES and discovering new treatment options. However, significant gaps in our understanding of the condition persist, necessitating further research to enhance HIES diagnosis and management. Through continued efforts in research and treatment, we aim to enhance the quality of life for individuals affected by HIES and ultimately discover a cure.

This case report is unique and adds to the existing literature as it highlights that since HIES is a rare diagnosis, it could be missed in patients especially those belonging to an under-resourced area. Our patient was diagnosed at 2 years of life and awareness amongst physicians and early diagnosis could have aided in improving his quality of life. Additionally, with newer treatment options now available the patients could be offered the option of a bone marrow transplant or stem cell therapy if a suitable donor is available. The work has been reported in line with the SCARE 2023 criteria^12^.

## Ethical approval

The ethical approval was not required for the case report as per the country’s guidelines.

## Consent

Written informed consent was obtained from the patient for publication and any accompanying images. A copy of the written consent is available for review by the Editor-in-Chief of this journal on request.

## Sources of funding

None.

## Author contribution

All the authors contributed equally in drafting, editing, revising and finalizing the case report.

## Conflicts of interest disclosure

None.

## Research registration unique identifying number (UIN)

Name of the registry: Not Applicable. Unique Identifying number or registration ID: Not Applicable. Hyperlink to your specific registration (must be publicly accessible and will be checked): Not Applicable.

## Guarantor

Fatema Ali Asgar Tashrifwala.

## Data availability statement

It will be available upon reasonable request to the corresponding author.

## Provenance and peer review

Not commissioned, externally peer-reviewed.
